# Pore Structural Features of Granite under Different Temperatures

**DOI:** 10.3390/ma14216470

**Published:** 2021-10-28

**Authors:** Hongmei Gao, Yongwei Lan, Nan Guo

**Affiliations:** 1 School of Civil Engineering, Northeast Forestry University, Harbin 150040, China; 2005801019@usth.edu.cn; 2 School of Architecture and Civil Engineering, Heilongjiang University of Science and Technology, Harbin 150022, China; 3 College of Mining Engineering, Heilongjiang University of Science and Technology, Harbin 150022, China; 2005800164@usth.edu.cn

**Keywords:** mercury injection experiment, scanning electron microscope (SEM), capillary pressure curve, median pore-throat ratio, median saturation pressure, median pore–throat radius, pore volume, pore size distribution, porosity, modified Winland model

## Abstract

To explore the effects of thermal actions on the pore structural features of granite, scanning electron microscope (SEM) and mercury injection experiments were carried out on granite after thermal treatment (25 °C to 400 °C). The pore structure was investigated from various perspectives, including the capillary pressure curve, the pore–throat ratio, the median saturation pressure, the median pore–throat radius, the porosity, the pore volume, and the pore size distribution. Based on mercury intrusion test data, the Winland model of permeability prediction was modified for a high-temperature tight granite reservoir. The results showed that: (1) As the temperature rose, the mercury injection curve was gradually flattened, and the mercury ejection efficiency gradually increased. Meanwhile, the pore–throat ratio and the median saturation pressure decreased exponentially, and the pore connectivity was enhanced. (2) The median pore–throat radius and the porosity of granite increased exponentially as the temperature increased. Above 200 °C, the median pore–throat radius and the porosity increased substantially. (3) The pore volumes of the transitional pores, mesopores and macropores, and the total pore volume inside the granite, increased as the temperature rose. Especially above 200 °C, the transitional pores and the mesopores were prominently developed, and the pore volumes of the transitional pores and the mesopores took up a significantly greater proportion of the total pore volume. (4) As the temperature rose, the pore size distribution of granite became more extensive, the pore–throat structure was obviously developed, and the pore–throat connectivity was enhanced. (5) The relationship between the micropores’ characteristic parameters and the macro-permeability in engineering was established though a modified Winland model, and the modified Winland model had a better prediction effect. The findings provide a solid basis for rock geothermal mining projects and related geotechnical engineering.

## 1. Introduction

Recently, geothermal energy has been favored by governments around the world as a clean energy source. It is generally believed that geothermal resources with economic development and utilization value are contained in the strata of rocks about 10 km beneath the surface, where the temperature can reach above 400 °C [1]. China is distributed with abundant geothermal resources, which are mainly stored in granite rock mass [2]. Therefore, the physicomechanical properties of granite at high temperatures are a topic of interest among researchers today [3].

It is well known that granite is a porous medium with a large number of pore-throats irregularly distributed across scales. Under thermal actions, pore-throats are produced, expand, and develop into new pore structures inside the rocks. These microscopic changes in the pore structure cause significant changes in the physicomechanical properties of rocks at the macroscopic level. Therefore, a quantitative description of the pore-throat structural features of rocks is the basis for establishing the correlation between microscopic pore-throat features and the macroscopic physicomechanical properties of the rocks [4].

Researchers have conducted a lot of studies on the evolution of the microscopic pore structures of rocks, and fruitful results have been achieved worldwide. Xu et al. [5], Wu et al. [6], and Gao et al. [7] employed a micropore structural analyzer and acoustic emission detector to investigate the micropore structural changes of granite under thermal actions, and determined the direct relationship between rock strength and micropore structural features. Loucks et al. [8] and Nie et al. [9] used high-resolution scanning electron microscopy to observe and describe the pore morphology of dense rocks. Allan et al. [10] used a scanning electron microscope to qualitatively describe the changes of the pore structure in shale, which were related to the pyrolysis products that escaped the rock. Bu et al. [11] and Xi et al. [12] performed liquid nitrogen adsorption tests on shale and used the pore fractal theory of rock to calculate the fractal dimension of the pore structure of shale. Chen et al. [13] and Tsakiroglou et al. [14] used the mercury injection method to perform fractal fitting on pores of various sizes in shale, and calculated their fractal dimension. Yang et al. [15] applied mercury injection to characterize pore structure during pyrolysis. Liu et al. [16] analyzed the evolution mechanism of the pore structures in shale under thermal actions. Zhao et al. [17] used a high-precision computed tomography (CT) test system to conduct three-dimensional microscopic observations of ruptures in granite at high temperatures, revealing that the crystal particles of granite have irregular spatial structures. Bai et al. [18] and Geng et al. [19] combined mercury intrusion porosimetry (MIP) and CT to analyze the pyrolysis characteristics and the pore structural characteristics of rocks. Ekberg et al. [20], Shang et al. [21], Yin et al. [22], Farquharson et al. [23], Jia et al. [24], Zhang et al. [25] and Xia et al. [26] studied the relationship between the internal pore structure and physical and mechanical properties of rocks after high-temperature heat treatment.

At present, the relevant research results are mainly focused on the qualitative study of the micro-fracture characteristics of high-temperature rock masses. The quantitative study of the micro-fracture characteristics of high-temperature rock masses still needs further work, and the application of the pressurized mercury injection method in the geothermal mining project at Daqing City is very rare. There are few reports on the pore and throat distribution characteristics of granite in the geothermal field of the Songnen Basin as assessed by mercury injection.

For these reasons, the dense granite collected from the Songnen Basin geothermal field (Daqing section) in Heilongjiang Province, China was taken as the subject in this paper. Scanning electron microscope (SEM) and mercury injection were used to test granite samples after thermal treatment. Various aspects, including the capillary pressure curve, the pore–throat ratio, the median saturation pressure, the median pore–throat radius, the porosity, the pore volume, and the pore size distribution were investigated. The evolution of granite pore structure under high temperature was studied using multiple parameters and angles, and the empirical formula between temperature and micro-parameters was obtained from the experimental data. Based on SEM and mercury injection test data, the permeability prediction Winland model was modified for a high-temperature and tight granite reservoir, the relationship between micropore characteristic parameters and macro-permeability in engineering was established though the modified Winland model, and the modified Winland model was shown to have a better prediction effect. At the same time, the research results have practical significance for the application of a granite reservoir in the geothermal field of Songnen Basin. The findings would provide a solid basis for rock geothermal mining and related geotechnical engineering projects.

## 2. Materials and Methods

**Figure 1:**
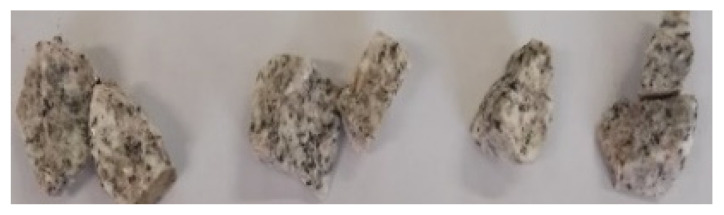
Granite sample.

The studied samples of granite rock mass were collected from the Songnen Basin geothermal field (Daqing section) in Heilongjiang Province, China. Fresh and with a fine texture, granite samples were processed into 0.5 cm × 0.5 cm × 0.5 cm test pieces as shown in Figure 1. In the process of sample preparation, we tried to ensure that the fracture development degree of each rock sample was basically the same. Then, the wave velocity of the test block was measured using the dynamic mode instrument, and the test block with a similar wave velocity was selected to reduce the test error. Five temperature points were selected at 25 °C, 100 °C, 200 °C, 300 °C, and 400 °C, respectively. The rock samples were heated by the automatic electronic muffle furnace at 5 °C/min to the specified temperatures (100 °C, 200 °C, 300 °C, and 400 °C). The temperatures were kept for five hours and then slowly lowered to room temperature.

Test principle of mercury injection instrument: Since the mercury is not infiltrative into rock solids, external pressure must be applied to inject mercury into the pores of rocks. The energy required to increase the pore volume of mercury injected into the solid rock is equal to the work done by the external force, and the pressure required should overcome the capillary force that drives the mercury to flow out of the pores. The smaller the pores, the greater the capillary force, and the higher the pressure required for mercury injection and ejection.

**Figure 2:**
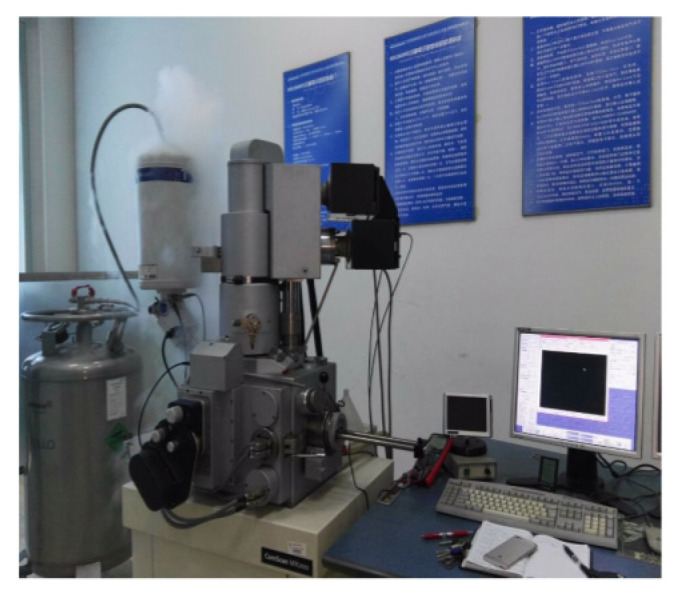
Scanning electron spectrometer.

**Figure 3:**
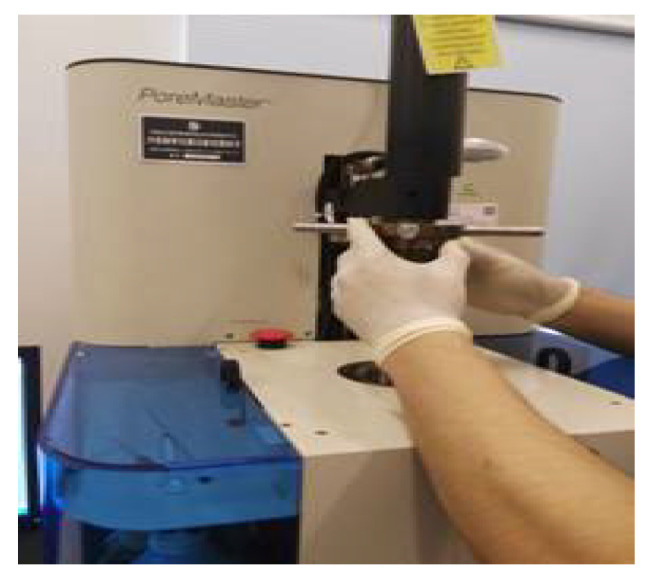
High-performance automatic mercury injection instrument.

A scanning electron spectrometer was used as shown in Figure 2. A high-performance automatic mercury injection instrument was used, as shown in Figure 3. The rock samples were dried and put into the core chamber of the automatic mercury injection instrument. The injection pressure was increased. When it became stable, the pressure and volume of the mercury injected were recorded. The capillary pressure curve, the porosity, the pore volume, and the pore size distribution curve could be obtained by constantly changing the mercury injection pressure. At the same temperature measurement point, three granite samples were taken for mercury injection testing, and the average value or a typical image of the test results were taken.

Using a cylindrical pore model, the pore volume was calculated according to the relationship between the pressure and the capacitance. The relationship between the specific surface area and pore–throat distribution can be fitted by the Washburn equation [14], whereby

(1) P = −2σcosa/r

where r denotes the pore–throat radius (nm), P denotes the capillary pressure ($10^{-14}\;{\mathrm{dyn}/\mathrm{nm}}^{2}$), a denotes the wetting contact angle of the mercury (°), and σ denotes the surface tension of the mercury ($10^{-14}\;{\mathrm{dyn}/\mathrm{nm}}^{2}$). In this study, the contact wetting angle of the mercury was 146° and the surface tension of the mercury was $4.80\;\times \;10^{-12}\;{\mathrm{dyn}/\mathrm{nm}}^{2}$, then we determined that

(2) P = 7.5/r

Seepage test: The heated samples were naturally cooled to room temperature, and the oil was washed with benzene and ventilated and dried for a period of time. The length and diameter of the specimen were measured. The self-developed low permeability rock testing system was used to conduct the gas permeability test via the quasi-static method, and the permeability was calculated by the Darcy formula.

## 3. Results and Analysis

### 3.1. Results of Scanning Electron Microscope (SEM)

**Figure 4:**
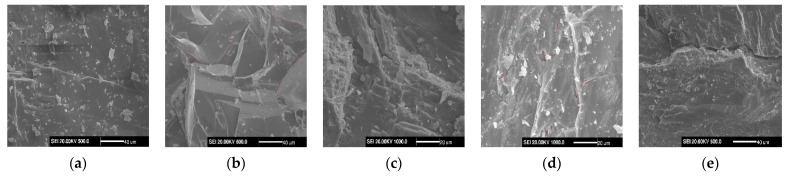
Scanning electron microscope characterization of samples: (**a**) 25 °C, (**b**) 100 °C, (**c**) 200 °C, (**d**) 300 °C, (**e**) 400 °C.

It can be seen from Figure 4 that the granite sample had uniform density, and its surface contained certain initial microcracks and holes (ink bottle pores), the shapes of which were irregular, reflecting the density of granite and the small pore throats. As regards the relationship between the pore throat characteristics of the complex, the pore connectivity was poor. With the increase in temperature, microcracks and holes in the granite were obviously developed.

At present, there is no unified standard for the classification of rock pores worldwide. According to the classification standards proposed by Chinese experts, the pores in granite were divided into four categories [27]: macropores (diameter > 1000 nm), mesopores (1000 nm ≥ diameter > 100 nm), transitional pores (100 nm ≥ diameter > 10 nm), and micropores (diameter ≤ 10 nm). 

### 3.2. Capillary Pressure Curve of Mercury Injection and Ejection

**Figure 5:**
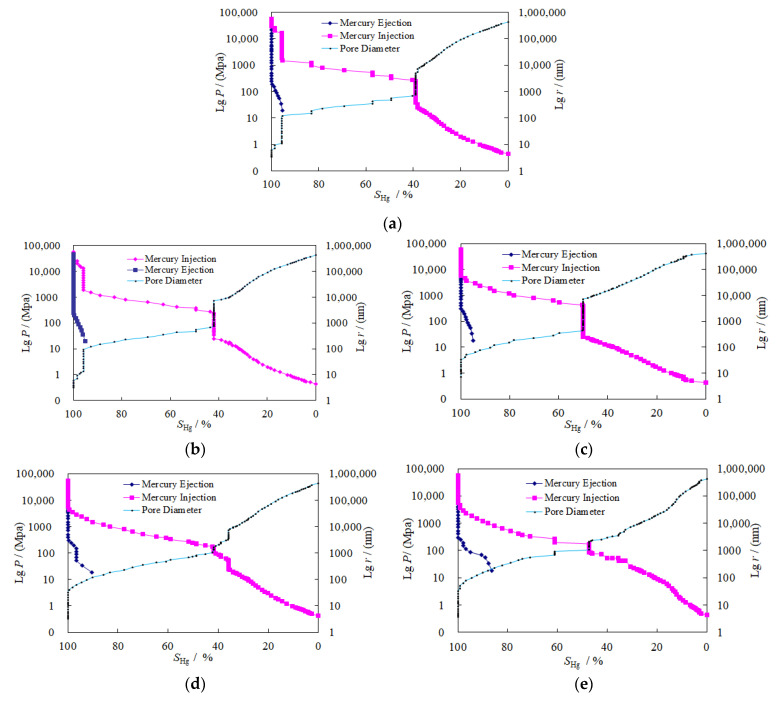
Capillary pressure curves and pore diameters of granite at different temperatures: (**a**) 25 °C, (**b**) 100 °C, (**c**) 200 °C, (**d**) 300 °C, (**e**) 400 °C.

The experimental data obtained from the mercury injection test are shown in Table 1.The capillary pressure curve reflects the development of the pores and throats and the connectivity between the pores. The resulting capillary pressure curve and pore diameter curve of granite are shown in Figure 5. 

The pore space (pore volume) inside the granite can be divided into the pore body and the pore throat parts. A throat part is a narrow area that connects the pores and controls the seepage capacity of the rock, while a pore body is a wider area of the pore space. As suggested by Figure 5, from 25 °C to 400 °C, the continuity of the mercury injection curve and the pore diameter curve of granite was gradually enhanced. At 25 °C, the mercury injection curve and pore diameter curve of granite underwent a sudden change (the mercury saturation remained unchanged, while the mercury injection pressure continued to rise) when the saturation reached 40% and 95%. The mercury injection curve and pore diameter curve are stepped and show discontinuities, indicating the concentrated pore size distribution, thin throats, and poor pore connectivity in granite. At 100 °C, a sudden change in the mercury injection curve and pore diameter curve of granite occurred when the mercury injection saturation reached 42% and 97%. At 200 °C, a sudden change in the mercury injection curve and pore diameter curve of granite occurred when the mercury injection saturation reached 50%. At 300 °C and 400 °C, there was no obvious sudden change in the mercury injection curve or pore diameter curve of granite, and the curve continuity was gradually enhanced. This indicates that as the temperature increased, the pores developed, the pore size distribution expanded, and the pore connectivity was enhanced.

According to Figure 5 and Table 1, the maximum mercury injection saturation (100%) reflects the sum of the proportions of the pore body and the pore throat parts in granite, indicating that all the pore spaces inside granite were infused with mercury, and the experimental accuracy was high. The mercury ejection saturation and the mercury ejection efficiency reflect the proportion of the throat parts, while the residual mercury saturation reflects that of the pore body in granite. At 25 °C, 100 °C, 200 °C, 300 °C, and 400 °C, the mercury ejection efficiency was 5.647%, 6.769%, 8.342%, 9.557%, and 16.786%, respectively. The mercury ejection efficiency slowly increased, and the residual saturation gradually decreased. This suggests that the proportion of throats in the pore spaces was gradually increasing, and the throats developed more obviously than the pores. In general, the mercury ejection curve of granite was short, and the mercury ejection efficiency was low. This suggests that the residual mercury was mainly in the “ink bottle” pores, with thin throats and large pore bodies, in granite.

### 3.3. Pore–Throat Ratio

**Figure 6:**
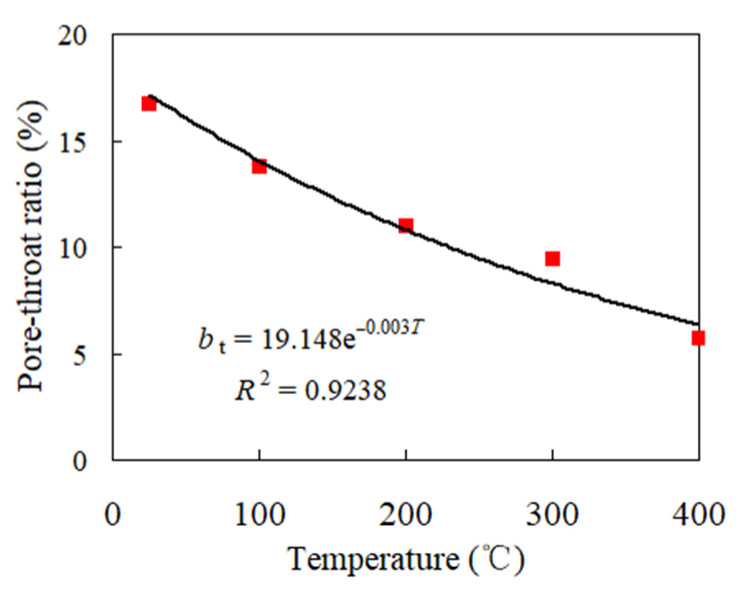
The pore–throat ratio of granite under different temperatures.

The pore–throat ratio is one of the key parameters for evaluating geothermal mining projects. Under different thermal actions, the pore–throat ratio of granite was determined by reference to Reference [28]. At 25 °C, 100 °C, 200 °C, 300 °C, and 400 °C, the pore–throat ratio of granite was 16.71, 13.77, 10.99, 9.46, and 5.76, respectively. On this basis, the curve of the changes in the pore–throat ratio with the temperature can be obtained, as shown in Figure 6. From 25 °C to 400 °C, the pore–throat ratio of granite gradually decreased, and the fitting function between the pore–throat ratio ($b_{t}$) and the temperature (T) is as follows:

(3) $$b_{t}\;=\;19.148e^{-0.003T}$$

According to Figure 6, at 100 °C, 200 °C, 300 °C, and 400 °C, the pore–throat ratio of granite decreased by 17.57%, 34.24%, 43.36%, and 65.51%, respectively, from that at 25 °C. From 25 °C to 200 °C, the pore–throat ratio was great, showing that the throat was underdeveloped with low permeability, which is not conducive to heat transfer and later mining in geothermal projects. From 300 °C to 400 °C, the pore–throat ratio was small, showing that the throat space of granite gradually increased, and the throat developed with increased seepage capacity, which is conducive to the transfer of geothermal heat through the throat.

### 3.4. Median Saturation Pressure and Median Pore–Throat Radius

**Figure 7:**
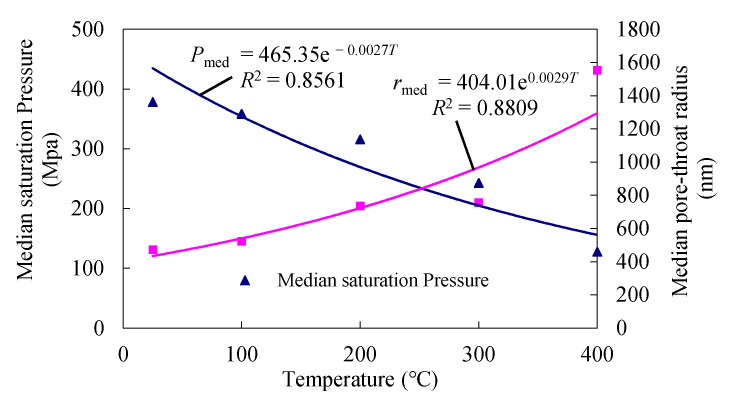
Median saturation pressure and median pore–throat radius curves of granite at different temperatures.

The median saturation pressure is the mercury injection pressure value (Figure 5) when mercury injection saturation reaches 50%. The median pore–throat radius is the radius of granite when the mercury injection saturation reaches 50%. From 25 °C to 400 °C, the median saturation pressure and median pore–throat radius at mercury saturation of granite are shown in Table 1. The curves of change in the median saturation pressure and median pore–throat radius with temperature are shown in Figure 7.

According to Table 1 and Figure 7, the median saturation pressure of granite decreased as the temperature increased, and the median pore–throat radius of granite increased as the temperature increased. The fitting functions for the median saturation pressure ($P_{\mathrm{med}}$), the median pore–throat radius ($r_{\mathrm{med}}$) and the temperature (T) are as follows:

(4) $$P_{\mathrm{med}}\;=\;465.35e^{-0.0027T}$$

*r*_med_ = 404.01e^0.0029*T*^ (5)

At 25 °C, the median saturation pressure and median pore–throat radius of granite were 378.12 MPa and 472.7 nm, respectively. At 100 °C, 200 °C, 300 °C, and 400 °C, the median saturation pressure of granite was 0.948, 0.835, 0.642, and 0.338 times of that at 25 °C, respectively. The median saturation pressure of granite decreased, and the median pore–throat radius of granite increased, indicating that the pores gradually developed and the pore connectivity was enhanced. The median saturation pressure of granite changed in a pattern similar to the pore–throat ratio.

At 100 °C and 200 °C, the median pore–throat radius was 1.106 and 1.555 times that at 25 °C, and the increasing trend was not obvious. At 300 °C and 400 °C, the median pore–throat radius was 1.595 and 3.285 times that at 25 °C, and the increasing trend was obvious. The changing pattern of the median pore–throat radius increased with temperature, which is consistent with the changing pattern of the median saturation pressure.

### 3.5. Porosity

The porosity before heat treatment is critical in the study. Therefore, in this paper, the mercury injection method and nuclear magnetic resonance method were used to measure the porosity of granite before heat treatment. Following mercury injection experiments, the porosity of granite at different temperatures is shown in Table 2. The porosity of granite before heat treatment was 0.835%, as assessed by nuclear magnetic resonance [29], and the average porosity before heat treatment was 0.842%, as assessed by mercury injection test. The porosity obtained by the two methods before heat treatment was very close, indicating the reliability of the test data.

**Figure 8:**
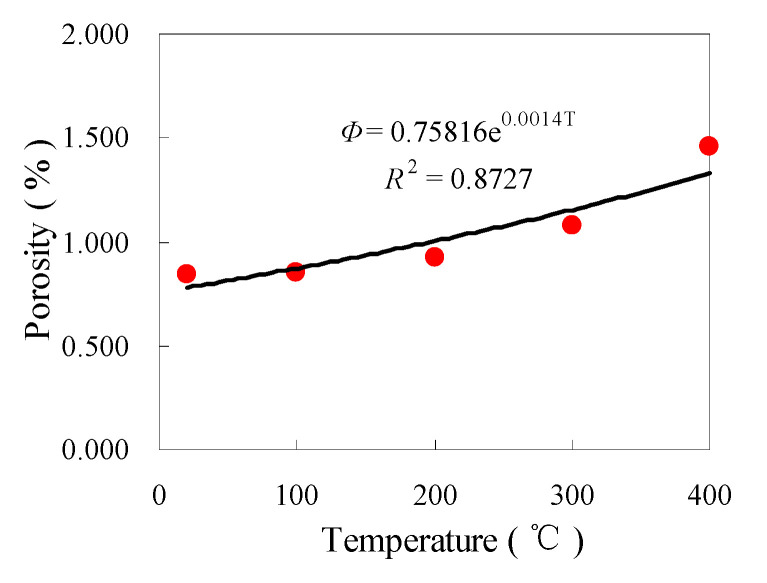
Porosity of granite under different temperatures.

As can be seen from Table 2, the measurement error of porosity is small and the measurement results are accurate. The curve of change in the porosity with temperature is shown in Figure 8. The porosity of granite increased as the temperature rose, which is consistent with previous findings [20,26]. The changes in the porosity (Φ) with temperature (T) can be expressed by an exponential function, as follows

Φ = 0.7581e^0.0014*T*^ (6)

As suggested by Figure 8, at 25 °C, the porosity of granite was 0.8424, which was low because granite is a dense type of rock. At 100 °C and 200 °C, the porosity of granite was 1.14 and 10.10 times greater than that at 25 °C, indicating that the thermal treatment caused a variety of physical changes in the rock structure, mainly due to the development of the micropores and macropores formed by the evaporation of water and the thermal expansion of minerals. At 300 °C and 400 °C, the porosity of granite was 27.69 and 72.78 times greater than that at 25 °C, indicating that the thermal treatment made granite water evaporate and the minerals thermally expand. As a result, thermal stress induced the development of macropores, mesopores, and transitional pores; moreover, pore–throats developed and the connectivity was enhanced. The results are consistent with those of SEM.

### 3.6. Pore Volume

**Figure 9:**
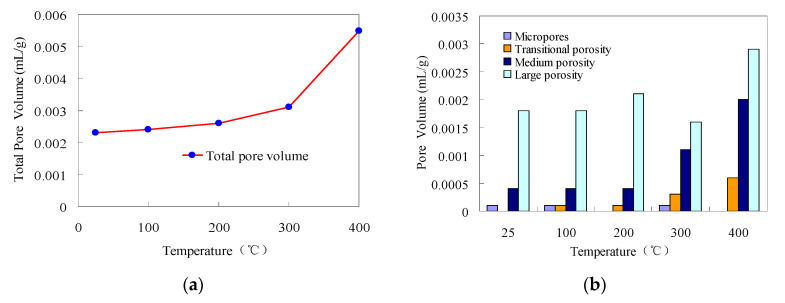
Pore volume of granite at different temperatures: (**a**) total pore volume, (**b**) pore volume of various pores.

According to the capillary pressure curve (Figure 5), the amount of injected mercury at each point when the mercury injection pressure reached equilibrium reflects the pore volume connected by the pore–throat radius. The pore volume was obtained through high-pressure mercury injection, as shown in Table 3. The changing pattern of the pore volume of granite with temperature is shown in Figure 9.

According to Table 3 and Figure 9a, from 25 °C to 400 °C, the total pore volume of granite increased as the temperature rose, which is consistent with previous findings [14]. At 100 °C and 200 °C, the total pore volume of granite was 4.348% and 13.043% greater than that at 25 °C, which are insignificant increases. At 300 °C and 400 °C, the pore volume of granite was 34.783% and 139.130% greater than that at 25 °C, which are significant increases.

According to Table 3 and Figure 9b, the pore volumes of the macropores, mesopores, transitional pores, and micropores inside granite varied slightly with the temperature. At 25 °C, 100 °C, and 200 °C, the pore volumes of the mesopores, transitional pores, and micropores changed insignificantly as the temperature increased, while the pore volume of the macropores and the total pore volume slowly increased as the temperature rose. At 300 °C and 400 °C, the volumes of the macropores, mesopores, and transitional pores and the total pore volume increased as the temperature rose. In particular, the pore volumes of the transitional pores and the mesopores showed the most prominent increasing trend, and the increasing gradient was great.

According to Table 3 and Figure 9, as the temperature rose, the proportions of the pore volumes of macropores, mesopores, transitional pores, and micropores in the total pore volume changed. From 200 °C to 400 °C, the proportion of the pore volume of transitional pores increased from 3.846% to 10.909%, while that of the mesopores increased from 15.385% to 36.364%, indicating that the distribution range of mesopores was well developed with a strong sensitivity to the temperature, and the connectivity was enhanced above 200 °C.

### 3.7. Pore Size Distribution

**Figure 10:**
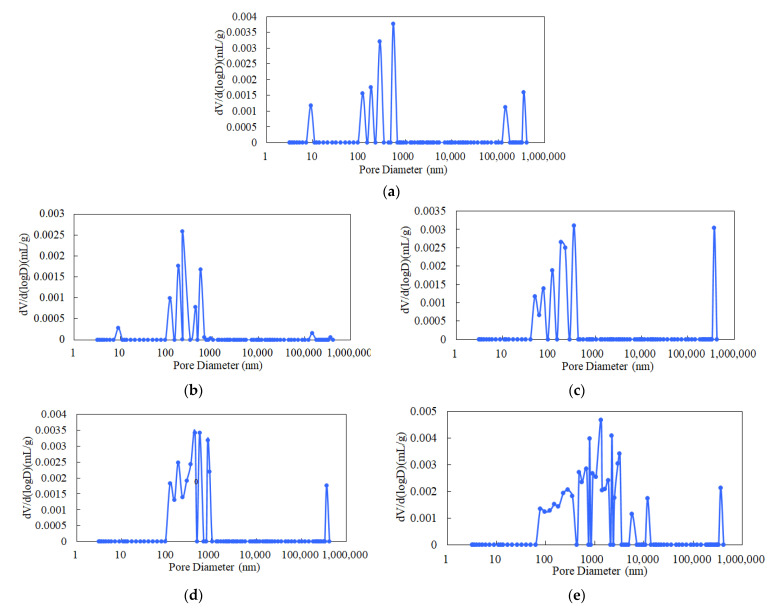
Pore size distribution of granite at different temperatures: (**a**) 25 °C, (**b**) 100 °C, (**c**) 200 °C, (**d**) 300 °C, (**e**) 400 °C.

Using Equation (2), the pore–throat radius at each level could be obtained. The pore size distribution of granite was obtained through mercury injection experiments, as shown in Figure 10.

As suggested by Figure 10, at 25 °C, the pore size of granite showed multiple discontinuous peaks between 70 and 720 nm, and the minimum pore size was 8.9 nm, indicating that the granite was a dense rock with poor pore connectivity at this point. At 100 °C and 200 °C, the pore size distribution curve still showed multiple peaks. The pore size showed multiple continuous peaks between 70 and 780 nm, and the minimum pore sizes were 9.3 nm and 50.2 nm, respectively. At 300 °C, the pore size distribution curve still showed multiple peaks. The pore size showed multiple continuous peaks between 100 and 980 nm, and there were more multi-peak points than at 200 °C. The pore connectivity gradually increased. At 400 °C, the pore size distribution curve still showed multiple peaks. The pore size showed multiple continuous peaks between 70 and 10,000 nm, and there were more multi-peak points than at 300 °C. The distribution range of the pore radius increased, and the pores gradually developed more obviously. As a result, the pore connectivity continued to increase, and the pore connectivity increased significantly.

In summary, the changing mechanisms of granite shape, the capillary pressure curve, the pore–throat ratio, the median saturation pressure, the median pore–throat radius, the porosity, the pore volume, and the pore size distribution associated with temperature were unified. The findings are mutually corroborating, indicating the validity of the experimental results.

## 4. Modification of Permeability Prediction Model

At present, there are many permeability models used to predict the permeability of rock, but there are few models used to predict the permeability of high-temperature granite, and so the parameters of a typical permeability prediction model were modified.

Winland model [30]:

lg*R*_35_ = 0.732 + 0.588lg*k* − 0.864lg Φ (7)

where *k* denotes the permeability (m^2^), and *R*_35_ denote the corresponding pore–throat radius (μm), when the mercury saturation is 35%. Φ denotes the porosity at granite under different temperatures.

The Winland model takes *R*_35_ as the characteristic pore–throat radius. In this paper, the permeability of granite at high temperatures was predicted by data regression analysis (*R*_10_, *R*_20_, *R*_25_, *R*_30_, *R*_35_, *R*_40_ and *R*_50_), and *R*_25_ was determined as the characteristic pore–throat radius.

**Figure 11:**
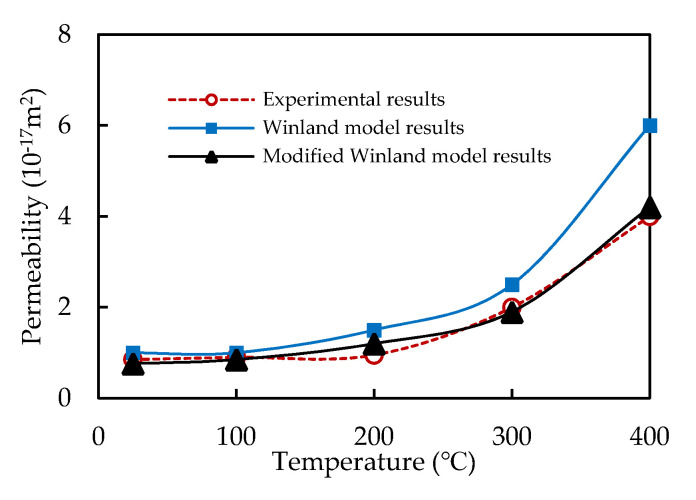
Permeability of granite under different temperatures.

Based on mercury intrusion test data, the modified Winland model could predict permeability through porosity and pore–throat radius. The permeability of the granite at high temperatures was as shown in Figure 11. As suggested by Figure 11, the modified permeability prediction values were closer to the measured permeability value, and the errors were smaller. The modified Winland model was more suitable for the permeability prediction of granite at a high temperature.

## 5. Conclusions

Based on the scanning electron microscope (SEM) and mercury injection method, the pore structure evolution of granite was studied after thermal treatment (25 °C to 400 °C). Based on mercury intrusion test data, the Winland model of permeability prediction was modified for a high-temperature tight granite reservoir. The conclusions are as follows:

(1) As the temperature rose, microcracks and holes obviously developed in granite, as shown by the electron microscopic scanning results: the mercury injection curves were gradually flattened, and the mercury ejection efficiency gradually increased. The pore–throat ratio and the median saturation pressure decreased exponentially as the temperature increased;
(2) The median pore–throat radius and the porosity of granite increased exponentially as the temperature rose. Above 200 °C, the median pore–throat radius and the porosity increased faster;
(3) The pore volumes of the transitional pores, mesopores, and macropores and the total pore volume of granite generally increased as the temperature rose. Above 200 °C, various pores developed. Among them, the transitional pores and the mesopores were more prominent, taking up a significantly greater proportion in the total pore volume;
(4) As the temperature rose, the pore size distribution of granite expanded, and the pores developed more obviously. In particular, above 200 °C, the pore size distribution and the pore connectivity were significantly increased;
(5) The modified permeability prediction values were closer to the measured permeability value. The modified Winland model was more suitable for the permeability prediction of granite at high temperatures.

## Figures and Tables

**Table 1:** Experimental results.

Temperature T (°C)	$\mathrm{Residual}\;\mathrm{Mercury}\;\;\mathrm{Saturation}\;S_{R}(\%)$	$\mathrm{Maximum}\;\mathrm{Mercury}\;\;\mathrm{Injection}\;\mathrm{Saturation}\;S_{\max}(\%)$	Mercury Ejection Saturation (%)	$\mathrm{Mercury}\;\;\mathrm{Ejection}\;\mathrm{Rate}\;\;(S_{\max}-S_{R})/S_{\max}(\%)$	$\mathrm{Median}\;\;\mathrm{Saturation}\;\;\mathrm{Pressure}\;P_{med}\;(\mathrm{Mpa})$	$\mathrm{Median}\;\;\mathrm{Pore}–\mathrm{Throat}\;\;\mathrm{Radius}\;r_{med}\;(\mathrm{nm})$	Porosity Φ (%)
25	94.353	100	5.647	5.647	378.12	472.70	0.842
100	93.231	100	6.769	6.769	358.45	523.00	0.852
200	91.658	100	8.342	8.342	315.78	734.90	0.928
300	90.443	100	9.557	9.557	242.90	754.00	1.076
400	85.214	100	16.786	16.786	127.73	1552.60	1.456

**Table 2:** Porosity of granite at different temperatures.

Temperature T(°C)	Porosity of Three Samples Φ(%)	$\mathrm{Average}\;\mathrm{Porosity}\;\Phi_{\mathrm{Ave}}(\%)$	Error Analysis *E* = (Φ − Φ*_Ave_*)/Φ*_Ave_* (%)
25	0.812, 0.871, 0.860	0.842	−3.56, 3.44, 2.14
100	0.829, 0.889, 0.839	0.852	−2.70, 4.34, −1.53
200	0.921, 0.919, 0.945	0.928	−0.75, −0.97, 1.83
300	1.096, 1.011, 1.119	1.076	1.86, −6.04, 4.00
400	1.510, 1.397, 1.462	1.456	3.71, −4.05, 0.41

**Table 3:** Pore volume of granite at different temperatures.

Temperatures (°C)	Volume of Diffrent-Sized Pores/(mL/g)					Proportion of the Pore Volume of Various Pores/(%)			
	$\mathrm{Total}\;\mathrm{Pore}\;\;\mathrm{Volume}\;V_{0}$	$\mathrm{Micropores}\;V_{1}$	$\mathrm{Transitional}\;\;\mathrm{Pores}\;V_{2}$	$\mathrm{Mesopores}V_{3}$	$\mathrm{Macropores}\;V_{4}$	$V_{1}/V_{0}$	$V_{2}/$ $V_{0}$	$V_{3}/$ $V_{0}$	$V_{4}/$ $V_{0}$
25	0.0023	0.0001	0.0000	0.0004	0.0018	4.348	0.000	17.391	78.261
100	0.0024	0.0001	0.0001	0.0004	0.0018	4.167	4.167	16.667	75.000
200	0.0026	0.0000	0.0001	0.0004	0.0021	0.000	3.846	15.385	80.769
300	0.0031	0.0001	0.0003	0.0011	0.0016	3.226	9.677	35.484	51.613
400	0.0055	0.0000	0.0006	0.0020	0.0029	0.000	10.909	36.364	52.727

Note: $V_{1}/V_{0}$ denotes the percentage taken up by the pore volume of the micropores in the total pore volumes, $V_{2}/$ $V_{0}$ denotes the percentage of the transitional pores in the total pore volumes, $V_{3}/$ $V_{0}$ denotes the percentage taken up by the pore volume of the mesopores in the total pore volumes, and $V_{4}/$ $V_{0}$ denotes the percentage taken up by the pore volume of the macropores in the total pore volumes.

## Data Availability

Data sharing is not applicable to this article.
